# In this month’s Bulletin

**DOI:** 10.2471/BLT.16.000116

**Published:** 2016-01-01

In the editorial section, Joseph Kutzin & Susan P Sparkes (2) define universal health coverage, health systems strengthening and health security and resilience. Samy A Alsirafy & Dina E Farag (3) argue that immediate action is needed to make oral morphine available in Egypt. 

In the news section, Dale Gavlak reports on how mental health services are becoming more available to the Syrian population in spite of the crisis (6–7). Charles Mgone talks to Fiona Fleck about building research partnerships (8–9).

## Brazil

### Folic acid in flour

Leonor Maria Pacheco Santos et al. (22–29) assess if folic acid fortification has contributed to a reduction in neural tube defects. 

## Burkina Faso 

### Preventing schistosomiasis 

Hamado Ouedraogo et al. (37–45) evaluate the impact of a decade of preventive chemotherapy in school-age children. 

## China

### Better mortality data 

Shiwei Liu et al. (46–57) describe the creation of a national system for mortality surveillance. 

## India 

### Improving vital statistics 

Mamta Gupta et al. (10–21) consider whether the civil registration system can be used as an accurate source of mortality data. 

## Nepal 

### Keeping doctors in remote hospitals 

Mark Zimmerman et al. (65–70) describe a staff support programme in rural hospitals.

## Thailand 

### Buying medical services abroad

Thinakorn Noree et al. (30–36) investigate the economic impact of medical tourism.

## Global 

### Improving research design and reporting

Simon Hales et al. (58–64) propose reporting guidelines for implementation and operational research. 

### Pharmaceuticals in Africa

Jicui Dong & Zafar Mirza (71–72) encourage international development partners to foster local pharmaceutical production. 

### Telemedicine for astronauts

Alfred Papali (73–74) uses the international space station to stress the feasibility of providing healthcare in remote areas. 

